# Dual Beneficial Effects of α-Spinasterol Isolated from *Aster pseudoglehnii* on Glucose Uptake in Skeletal Muscle Cells and Glucose-Stimulated Insulin Secretion in Pancreatic β-Cells

**DOI:** 10.3390/plants11050658

**Published:** 2022-02-28

**Authors:** Dahae Lee, Ji-Young Kim, Hak Cheol Kwon, Jaeyoung Kwon, Dae Sik Jang, Ki Sung Kang

**Affiliations:** 1 Cooperative-Center of Natural Product Central Bank for Biological Evaluation, College of Korean Medicine, Gachon University, Seongnam 13120, Korea; pjsldh@gachon.ac.kr; 2 Department of Biomedical and Pharmaceutical Sciences, Graduate School, Kyung Hee University, Seoul 02447, Korea; k_christina@khu.ac.kr; 3 KIST Gangneung Institute of Natural Products, Korea Institute of Science and Technology (KIST), Gangneung 25451, Korea; hkwon@kist.re.kr (H.C.K.); kjy1207@kist.re.kr (J.K.)

**Keywords:** *Aster pseudoglehnii*, α-Spinasterol, glucose-stimulated insulin secretion, glucose uptake

## Abstract

Herein, we determined whether α-Spinasterol, a stigmastane-type phytosterol isolated from *Aster pseudoglehnii*, potentially impacts glucose uptake and glucose-stimulated insulin secretion in skeletal muscle cells and pancreatic β-cells, respectively. We observed that *A. pseudoglehnii* and its fractions enhanced glucose uptake, with no toxic effects on C2C12 cells, with the *n*-hexane fraction exhibiting the most potent effect. α-Spinasterol, isolated from the *n*-hexane fraction, enhanced glucose uptake with no toxic effects on C2C12 cells. Additionally, α-Spinasterol increased the expression of associated proteins, including insulin receptor substrate-1, AMP-activated protein kinase, and glucose transporter type 4, as determined by Western blotting. Furthermore, α-Spinasterol enhanced insulin secretion in response to high glucose concentrations, with no toxic effects on INS-1 cells; this effect was superior to that demonstrated by gliclazide (positive control), commonly prescribed to treat type 2 diabetes (T2D). α-Spinasterol enhanced the expression of associated proteins, including insulin receptor substrate-2, peroxisome proliferator-activated receptor γ, and pancreatic and duodenal homeobox 1, as determined using Western blotting. The insulin secretory effect of α-Spinasterol was enhanced by a K^+^ channel blocker and L-type Ca^2+^ channel agonist and was suppressed by a K^+^ channel activator and L-type Ca^2+^ channel blocker. α-Spinasterol isolated from *A. pseudoglehnii* may improve hyperglycemia by improving glucose uptake into skeletal muscle cells and enhancing insulin secretion in pancreatic β-cells. Accordingly, α-Spinasterol could be a potential candidate for anti-T2D therapy.

## 1. Introduction

Type 2 diabetes (T2D) is a steadily growing metabolic disease worldwide, characterized by a decline in insulin secretion from pancreatic β-cells and impaired insulin function in insulin-target tissues, such as skeletal muscle [1]. Skeletal muscle is a glucose-metabolic organ, where 70–80% of insulin-dependent glucose uptake reportedly occurs after glucose intake [2]. During the pathogenesis of T2D, insulin resistance in skeletal muscles is considered one of the main characteristics. A previous study has revealed that limonene isolated from citrus fruits could induce glucose uptake via the Akt pathway in C2C12 skeletal muscle cells [3]. Myrtenal in pepper, eucalyptus, cumin, and mint has been shown to induce glucose uptake by upregulating Akt and glucose transporter type 4 (GLUT-4) in C2C12 cells [4,5]. Tricin, found in cereals, can reportedly induce glucose uptake by upregulating insulin receptor substrate-1 (IRS-1) and Akt [6]. Based on the above literature, Akt, GLUT-4, and IRS-1 play a central role in enhancing glucose uptake in skeletal muscle cells.

A decline in insulin secretion from pancreatic β-cells has also been confirmed as an important characteristic in the pathogenesis of T2D [7]. Glucose-stimulated insulin secretion (GSIS) is characteristic of differentiated pancreatic β-cells [8]. Robust GSIS after food consumption suppresses hyperglycemia. In particular, a decline in GSIS under insulin-resistant conditions may accelerate the transition to T2D [9]. A previous study has found that hypoxylonol F isolated from *Annulohypoxylon annulatum* increased GSIS by upregulating peroxisome proliferator-activated receptor γ (PPARγ) in the rodent insulin-secreting β-cell line, INS-1 cells [10]. In addition, *Panax ginseng* berry extracts can reportedly increase GSIS by upregulating insulin receptor substrate-2 (IRS-2) and pancreatic and duodenal homeobox 1 (PDX-1) in INS-1 cells [11]. Given the accumulated evidence, PPARγ, IRS-2, and PDX-1 appear to play a central role in improving GSIS in insulin-secreting β-cells. Despite the presence of a long list of antidiabetic drugs in the pharmaceutical market, the search for plant-derived natural products is still needed. Because T2D drugs have limitations in that they have unresolved side effects, plant-derived drugs are needed [12]. Although the antidiabetic effects of several natural products have been reported, limited reports are available regarding natural products that exhibit dual activities on both glucose uptake in insulin target cells and GSIS in insulin-secreting cells.

*Aster pseudoglehnii* is a perennial plant endemic to Korea and native to Ulleung Island. The young leaves and stems of *A. pseudoglehnii* are widely consumed in Korean cuisines. This plant has been used as a cold antipyretic, antitussive, and expectorant for tonsillitis [13]. It has been reported that *A. pseudoglehnii* has anti-adipogenic effects in vivo and in vitro [14] and ameliorates scopolamine-induced memory and cognitive impairment in mice [15]. However, the antidiabetic effects of *A. pseudoglehnii* remain unexplored. Thus, we determined whether α-Spinasterol, a stigmastane-type phytosterol isolated from *A. pseudoglehnii*, potentially impacts glucose uptake and glucose-stimulated insulin secretion in skeletal muscle cells and pancreatic β-cells, respectively.

## 2. Results

### 2.1. Isolation and Identification of α-Spinasterol

**Figure 1:**
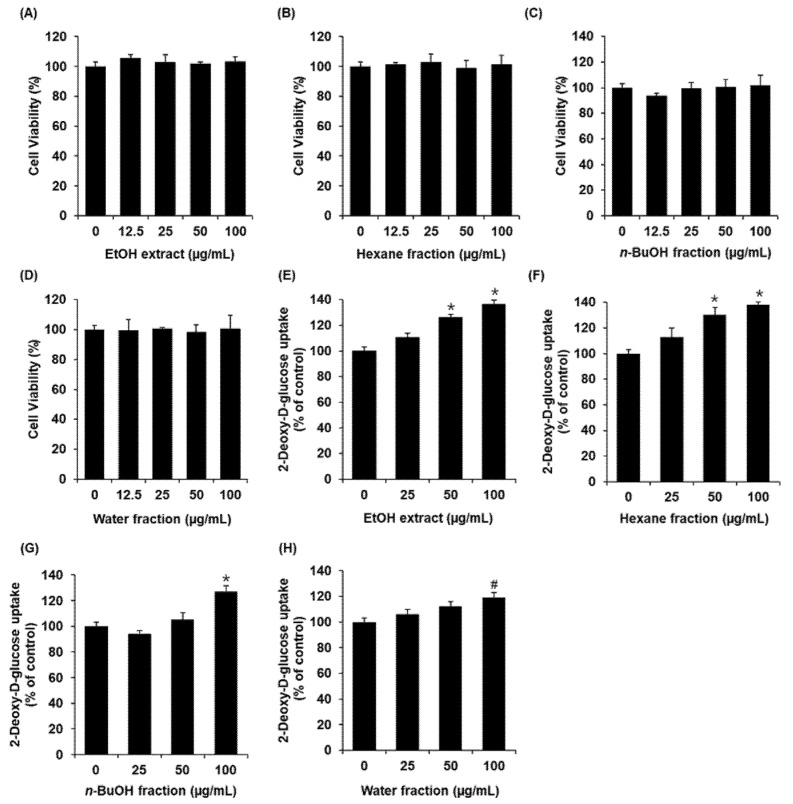
Effects of the EtOH extract of *Aster pseudoglehnii* and solvent fractions on the glucose uptake in C2C12 cells. MTT assay results of the cell viability of C2C12 cells after 24 h treatment with (**A**) EtOH extract, (**B**) *n*-hexane fraction, (**C**) *n*-BuOH fraction, and (**D**) water fraction, compared with the control (0 μM). Glucose uptake in C2C12 cells after 1 h incubation with (**E**) EtOH extract, (**F**) *n*-hexane fraction, (**G**) *n*-BuOH fraction, (**H**) water fraction, and 2-N-(7-Nitrobenz-2-oxa-1,3-diazol-4-yl) amino)-2-Deoxyglucose (2-NBDG), assessed by glucose uptake assay. Data represent the mean ± standard error of the mean (S.E.M.), n = 3, * *p* < 0.01, *^#^* *p* < 0.05 compared with the control.

A 70% EtOH extract of *A. pseudoglehnii* significantly enhanced glucose uptake in C2C12 cells (Figure 1). A bioassay-guided fractionation led to the isolation of an active compound from an *n*-hexane fraction of the 70% EtOH extract. The compound was isolated as a white powder and its liquid chromatography–electrospray ionization–mass spectrometry (LC–ESI–MS) data exhibited an [M+1]^+^ peak at *m*/*z* 413.0 (Figure S1). The ^1^H nuclear magnetic resonance (NMR) spectroscopic data of the compound displayed three doublet methyl signals at *δ*_H_ 1.02 (3H, d, *J* = 6.5 Hz, H-21), 0.84 (3H, d, *J* = 6.5 Hz, H-27), and 0.80 (3H, d, *J* = 6.0 Hz, H-26), and a triplet methyl proton at *δ*_H_ 0.81 (3H, t, *J* = 5.0 Hz, H-29), and two singlet methyl protons at *δ*_H_ 0.79 (3H, s, H-19), and 0.54 (3H, s, H-18) (Figure S2). Additionally, *trans*-olefinic protons at *δ*_H_ 5.14 (1H dd, *J* = 15.0, 8.5 Hz, H-22) and 5.02 (1H dd, *J* = 15.0, 8.5 Hz, H-23), an olefinic proton at *δ*_H_ 5.15 (1H, overlapped, H-7), and an oxygenated methine proton at *δ*_H_ 3.59 (1H, ddd, *J* =11.0, 6.5, 4.0 Hz, H-3) were detected. The ^13^C NMR exhibited 29 signals, including six methyl carbons (*δ*_C_ 21.5, 21.2, 19.1, 13.1, 12.3, and 12.1), four sp^2^ carbons, (*δ*_C_ 139.6, 138.3, 129.5, and 117.5), and an oxygenated sp^3^ carbon (*δ*_C_ 71.1) (Figure S3), inferring that this compound is a stigmastane-type phytosterol with two olefinic groups. The chemical structure of the compound was identified as α-Spinasterol by analysis of the MS and 1D NMR data and by comparison with those reported in the literature [16].

### 2.2. Effect of α-Spinasterol on Glucose Uptake in Skeletal Muscle Cells

All concentrations of the extract and fractions were non-toxic toward C2C12 cells (Figure 1A–D). We then confirmed whether the extract and fractions could increase glucose uptake activity at non-toxic concentrations. As shown in Figure 1E, the 70% EtOH extract of *A. pseudoglehnii* increased glucose uptake activity, presented as fold induction. The *n*-hexane fraction showed the most potent enhancing effect (Figure 1F).

**Figure 2:**
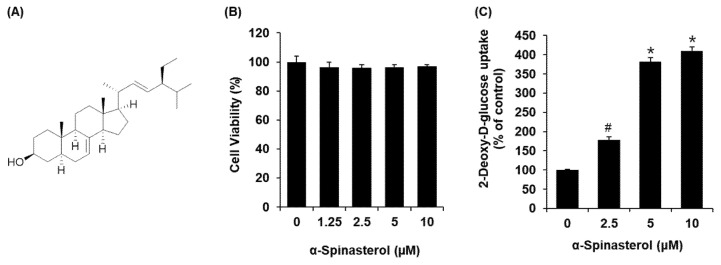
Effects of α-Spinasterol on the glucose uptake in C2C12 cells. (**A**) Chemical structure of α-Spinasterol; (**B**) MTT assay results of the cell viability of C2C12 cells after 24-h treatment with α-Spinasterol, compared with the control (0 μM); (**C**) Glucose uptake in C2C12 cells after 1 h incubation with α-Spinasterol and 2-N-(7-Nitrobenz-2-oxa-1,3-diazol-4-yl) amino)-2-Deoxyglucose (2-NBDG) assessed by glucose uptake assay. Data represent the mean ± standard error of the mean (S.E.M.), n = 3, * *p* < 0.01, *^#^* *p* < 0.05 compared with the control.

α-Spinasterol was isolated from the *n*-hexane fraction (Figure 2A). All examined α-Spinasterol concentrations were non-toxic toward C2C12 cells (Figure 2B). We then confirmed whether non-toxic α-Spinasterol concentrations could increase glucose uptake activity. As shown in Figure 2C, α-Spinasterol increased glucose uptake activity, presented as fold induction.

### 2.3. Effect of α-Spinasterol on the Protein Expression of P-IRS-1, IRS-1, P-AMPK, AMPK, and GLUT-4

**Figure 3:**
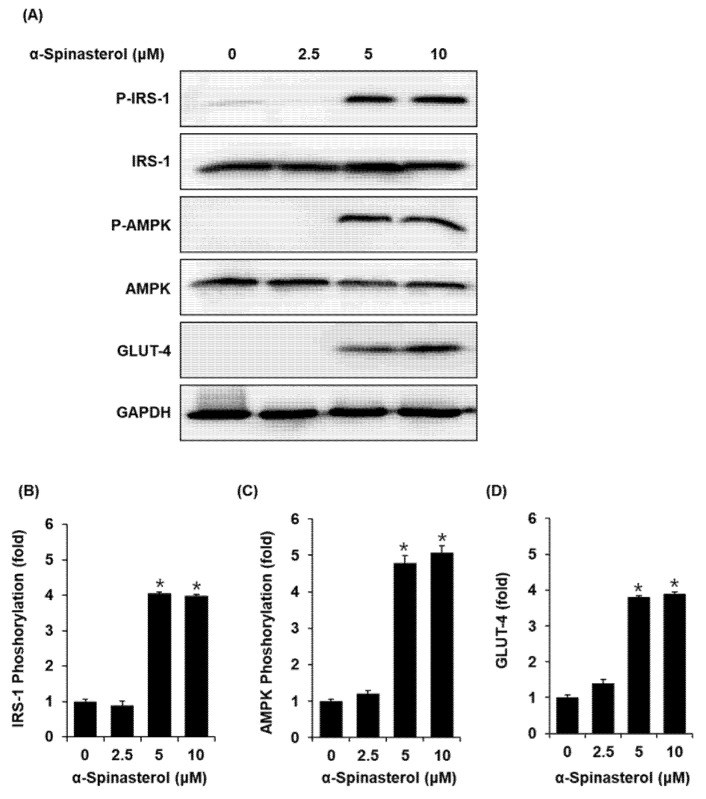
Effects of α-Spinasterol on the protein expression levels of phospho-insulin receptor substrate-1 (P-IRS-1), IRS-1, phospho-AMP-activated protein kinase (P-AMPK), AMPK, and glucose transporter type 4 (GLUT-4) in C2C12 cells. (**A**) Representative protein expression levels of P-IRS-1, IRS-1, P-AMPK, AMPK, GLUT-4, and glyceraldehyde 3-phosphate dehydrogenase (GAPDH) in C2C12 cells, treated or untreated with 2.5, 5, and 10 μM α-Spinasterol for 24 h; (**B**–**D**) Each bar graph presents the densitometric quantification of Western blot bands. Data represent the mean ± standard error of the mean (S.E.M.), n = 3, * *p* < 0.01 compared with the control.

Treatment with 5 and 10 μM α-Spinasterol increased protein expression of GLUT-4 and phosphorylation levels of IRS-1 and AMPK in C2C12 cells when compared with the untreated controls (Figure 3).

### 2.4. Effect of α-Spinasterol on GSIS

**Figure 4:**
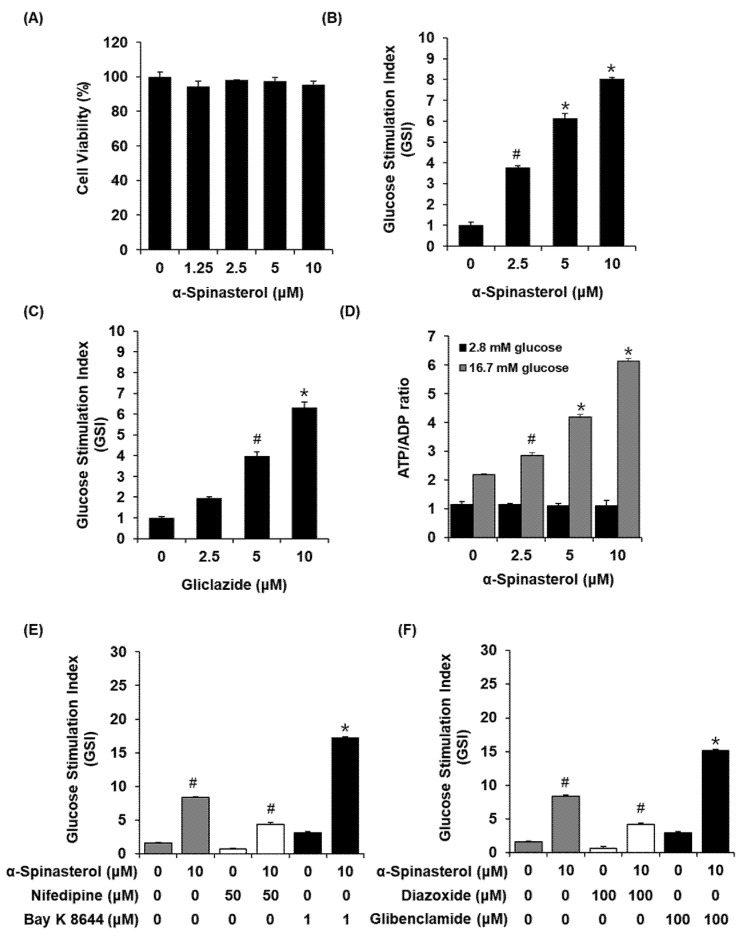
Effects of α-Spinasterol on glucose-stimulated insulin secretion (GSIS) in INS-1 cells. (**A**) MTT assay results on the cell viability of INS-1 cells after 24 h of treatment with α-Spinasterol when compared with the control (0 μM). Insulin secretion in INS-1 cells after 1 h incubation with basal (2.8 mM) and stimulant (16.7 mM) glucose concentrations in the presence or absence of (**B**) α-Spinasterol and (**C**) gliclazide (positive control), as assessed by the insulin secretion assay. Comparison of GSIS is expressed as fold stimulation in terms of the glucose-stimulated index (GSI, 16.7 mM glucose over 2.8 mM glucose for 1 h). (**D**) ATP/ADP ratio in INS-1 cells after 1 h incubation with basal (2.8 mM) and stimulant (16.7 mM) glucose concentrations in the presence or absence of α-Spinasterol, as assessed by the ADP/ATP ratio assay. (**E**) Insulin secretion in INS-1 cells after 1 h incubation with basal (2.8 mM) and stimulant (16.7 mM) glucose concentrations in the presence or absence of α-Spinasterol, nifedipine (L-type Ca^2+^ channel blocker), and Bay K 8644 (L-type Ca^2+^ channel activator), as assessed by insulin secretion assay. (**F**) Insulin secretion in INS-1 cells after 1 h incubation with basal (2.8 mM) and stimulant (16.7 mM) concentrations of glucose in the presence or absence of α-Spinasterol, diazoxide (K^+^ channel activator), and glibenclamide (K^+^ channel blocker), as assessed by the insulin secretion assay. Data represent the mean ± standard error of the mean (S.E.M.), n = 3, * *p* < 0.01, *^#^* *p* < 0.05 compared with the control.

α-Spinasterol was found to be non-toxic toward INS-1 cells (Figure 4A). As shown in Figure 4B, C, α-Spinasterol increased GSIS, expressed as the glucose-stimulated index (GSI). These values were superior to those of gliclazide (the positive control). These results suggested that α-Spinasterol enhanced insulin secretion in response to high glucose while exhibiting no toxic effects on INS-1 cells.

As shown in Figure 4D, α-Spinasterol increased the glucose-dependent ATP/ADP ratio. In addition, we evaluated the ability of α-Spinasterol to modulate K^+^ and Ca^2+^ channels. As shown in Figure 4E, F, α-Spinasterol-enhanced GSIS was enhanced by Bay K 8644 (L-type Ca^2+^ channel agonist) and glibenclamide (K^+^ channel blocker), whereas it was abrogated by nifedipine (L-type Ca^2+^ channel blocker) and diazoxide (K^+^ channel activator).

### 2.5. Effect of α-Spinasterol on the Protein Expression of P-IRS-2, IRS-2 (Ser731), PPARγ, and PDX-1

**Figure 5:**
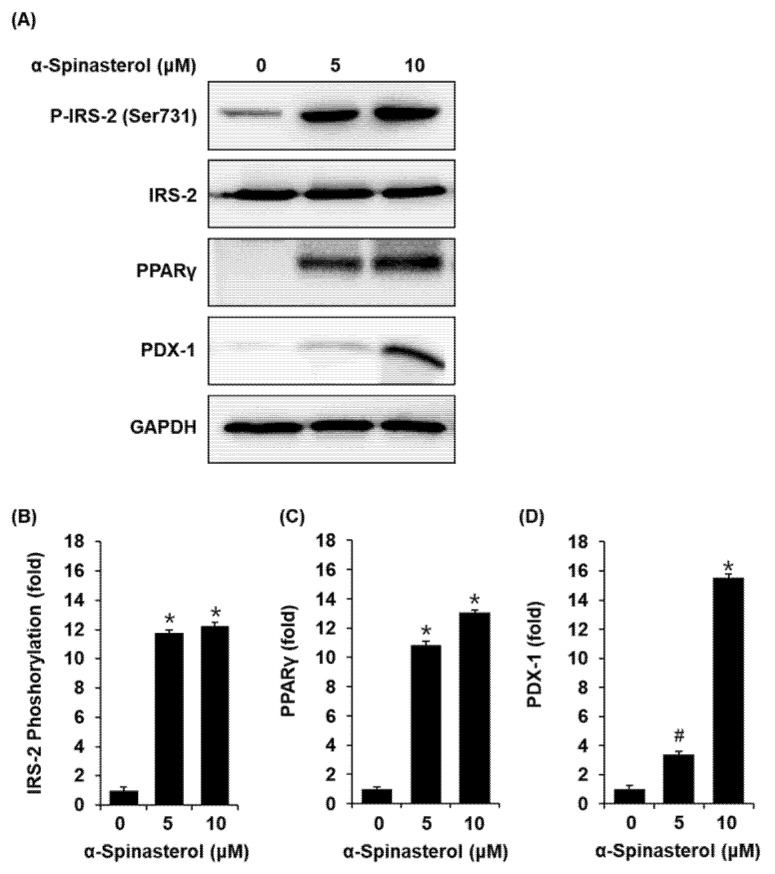
Effect of α-Spinasterol on the protein expression levels of phospho-insulin receptor substrate-2 (P-IRS-2) (Ser731), IRS-2, peroxisome proliferator-activated receptor γ (PPARγ), and pancreatic and duodenal homeobox 1 (PDX-1). (**A**) Representative protein expression levels of P-IRS-2 (Ser731), IRS-2, PPARγ, PDX-1, and glyceraldehyde 3-phosphate dehydrogenase (GAPDH) in INS-1 cells treated or untreated with 5 and 10 μM α-Spinasterol for 24 h. (**B**–**D**) Each bar graph presents the densitometric quantification of Western blot bands. Data represent the mean ± standard error of the mean (S.E.M.), n = 3, * *p* < 0.01, *^#^* *p* < 0.05 compared with the control.

**Figure 6:**
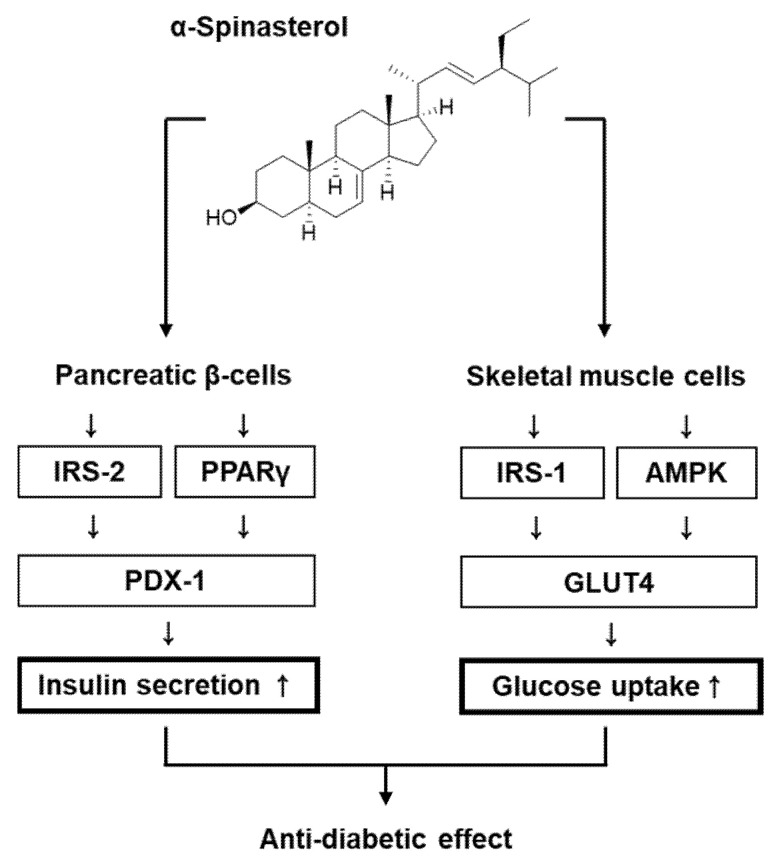
Schematic illustration of the effects of α-Spinasterol isolated from *A. pseudoglehnii* on glucose-stimulated insulin secretion in pancreatic β-cells and glucose uptake in skeletal muscle cells. IRS-1, insulin receptor substrate-1; IRS-2, insulin receptor substrate-2; AMPK, AMP-activated protein kinase; PDX-1, pancreatic and duodenal homeobox 1; GLUT-4, glucose transporter type 4; PPARγ, peroxisome proliferator-activated receptor γ.

Compared with the untreated controls, INS-1 cells treated with 5 and 10 μM α-Spinasterol showed increased protein expression of PPARγ, PDX-1, and phosphorylation levels of IRS-2 (Figure 5). Figure 6 presents a schematic illustration of the proposed mechanisms underlying the effects of α-Spinasterol on GSIS and glucose uptake in pancreatic β-cells and skeletal muscle cells, respectively.

## 3. Discussion

In the present study, our findings revealed that a 70% aqueous EtOH extract of the whole plants of *A. pseudoglehnii* significantly enhanced glucose uptake in C2C12 cells. Bioassay-guided isolation led to the isolation of α-Spinasterol as an active compound in the 70% EtOH extract. To the best of our knowledge, the presence of *α-Spinasterol* in *A. pseudoglehnii* is reported for the first time in this work. α-Spinasterol is a stigmastane-type phytosterol, which exhibits various pharmacological activities such as protective effects on diabetic nephropathy and benign prostatic hyperplasia [17,18], anti-inflammatory effects [18,19,20], and antibacterial effects [21]. However, the antidiabetic effects of α-Spinasterol remain unexplored. In addition, α-Spinasterol increased glucose uptake, which appeared to depend on the phosphorylation of IRS-1 and AMPK and increased GLUT-4 expression. In the postprandial state, skeletal muscle is the main site for glucose absorption, taking up glucose via an insulin-regulated glucose transporter such as GLUT-4. Thus, insulin-stimulated glucose uptake in the skeletal muscle depends on GLUT-4 expression at the plasma membrane [22]. Phosphorylation of IRS-1 and AMPK positively regulates the activation of GLUT-4 [23]. Therefore, upregulation of GLUT-4 in association with phosphorylation of IRS-1 and AMPK indicated that α-Spinasterol improved glucose uptake by translocating GLUT-4 to the skeletal muscle plasma membrane. In addition, we investigated the effect of α-Spinasterol on GSIS. We observed that α-Spinasterol increased GSIS, exhibiting a superior effect on GSI values than gliclazide (positive control), a drug commonly prescribed to treat T2D. Moreover, treatment with α-Spinasterol increased the ATP/ADP ratio. Glibenclamide and Bay K 8644 enhanced the α-Spinasterol-enhanced GSIS, which was suppressed by nifedipine and diazoxide. Thus, the increased ATP/ADP ratio indicated that the effect of α-Spinasterol on GSIS could be attributed to the closure of ATP-sensitive K^+^ (K_ATP_) channels and Ca2^+^ influx. It has been previously documented that an elevated ATP/ADP ratio plays an essential role in the closure of ATP-sensitive K^+^ (K_ATP_) channels and Ca2^+^ influx to promote insulin secretion [24]. Previous studies have reported that glibenclamide and Bay K 8644 stimulate insulin secretion in pancreatic β-cells. Conversely, diazoxide and nifedipine decrease insulin secretion in pancreatic β-cells [25,26,27,28].

Herein, INS-1 cells presented increased expression levels of IRS-2, PPARγ, and PDX-1 following treatment with α-Spinasterol. According to previous literature, treatment with PPARγ agonists can increase GSIS in mouse islets, rat islets, and INS-1 cells [29,30,31]. These results suggest that PPARγ activation could increase insulin secretion in pancreatic β-cells. Moreover, knockdown of IRS-2 and PDX-1 in mice has been shown to decrease GSIS [29,32,33]. Accumulating evidence suggests that upregulated protein expression of IRS-2, PPARγ, and PDX-1 may be responsible for an increase in GSIS in pancreatic β-cells. Therefore, improved GSIS after treatment with α-Spinasterol could be related to the increased protein expression of IRS-2, PPARγ, and PDX-1. α-Spinasterol isolated from *A. pseudoglehnii* may improve hyperglycemia by improving glucose uptake into skeletal muscle cells and enhancing insulin secretion in pancreatic β-cells. α-Spinasterol appears to be a promising, naturally-derived compound for treating T2D, warranting further preclinical investigation. In skeletal muscle, androgen receptor stimulation by 5α-dihydrotestosterone has been shown to cause an increase in glucose uptake in association with phosphorylation of GLUT-4 and Akt [34]. In pancreatic β-cells, activation of the androgen receptor and the estrogen receptor has been shown to cause an increase in GSIS [35,36]. Since α-Spinasterol is a sterol compound, these previous studies suggested the possibility that α-Spinasterol may exert an antidiabetic effect by acting on steroid nuclear receptors, including the androgen receptor and estrogen receptor. However, the α-Spinasterol action on the androgen receptor and the estrogen receptor has not been reported, thus future studies expanding on this study are needed. In addition, in vivo investigations, such as the oral glucose tolerance test, insulin sensitivity, gut sucrose content, gut perfusion, disaccharidase enzyme activity, and gut motility, are required to validate in vitro findings in Type 2 diabetes mellitus.

## 4. Materials and Methods

### 4.1. General Experimental Procedures

Open column chromatography was performed using silica gel (230–400 mesh ASTM, Merck, Darmstadt, Germany), and thin-layer chromatography analysis was performed on Kieselgel 60 F254 plates (silica gel, Merck, Darmstadt, Germany). The compound was visualized under UV light (254 and 365 nm) and 20% (*v*/*v*) H_2_SO_4_ reagent (Duksan, Gyeonggi-do, Korea). NMR spectra were obtained using a JNM-ECZ500R (JEOL, Tokyo, Japan) with deuterium solvent (Cambridge Isotope Laboratories, Tewksbury, MA, USA) as an internal standard, and chemical shifts were expressed as δ values. The LC–ESI–MS experiment was performed using Waters Acquity UPLC system and Waters Micromass Quattro Micro API (Waters, Milford, MA, USA) with Acquity UPLC BEH C18 column (2.1 × 50 mm i.d. 1.7 μm, Waters, Milford, MA, USA).

### 4.2. Plant Material

Whole plants of *Aster pseudoglehnii* Y. Lim, J. O. Hyun, and H. Shin (Compositae) were provided by Hantaek Botanical Garden (Yongin-Si, Gyeonggi-do, Korea) in May 2020. A voucher specimen (HTS2021-0001) was deposited at the Hantaek Botanical Garden (Yongin, Gyeonggi-do, Korea).

### 4.3. Extraction and Isolation

The whole plants of *A. pseudoglehnii* were dried for 3 days at 40 ± 2 °C in a dryer. The dried plant materials (966.5 g) were extracted with 70% aqueous EtOH (10 L, for 7 days) twice at room temperature. The solution was concentrated under reduced pressure to give a 70% EtOH extract (136.07 g). The extract was suspended in distilled water (1 L) and successively partitioned with *n*-hexane (1 L × 3) and *n*-butanol (1 L × 3) using a separatory funnel to yield *n*-hexane- (8.38 g), *n*-butanol- (43.28 g), and water-soluble fractions (70.35 g). The *n*-hexane soluble fraction showed the most potent enhancing effect in the glucose uptake assay. Thus, the active *n*-hexane-soluble fraction (7 g) was subjected to silica gel (70–230 mesh) column chromatography (CC, 4.8 × 44.5 cm) and eluted with *n*-hexane-EtOAc (8:2 to 0:10, *v*/*v*) to afford ten subfractions (H1–H10). Among the subfractions, H-4 exhibited significant activity in the glucose uptake assay. H4 (611 mg) was further fractionated using a flash chromatography system with Redi Sep-Silica (100 g, n-hexane: EtOAc, 9:1 to 6:4, *v*/*v*) to yield six fractions (H4-1–H4-6). *α*-Spinasterol (11.9 mg) was purified by recrystallization with MeOH from H4-3.

#### *α*-Spinasterol

White powder; ^1^H (500 MHz, chloroform-*d*) δ 5.15 (1H, overlapped, H-7), 5.14 (1H dd, *J* = 15.0, 8.5 Hz, H-22), 5.02 (1H dd, *J* = 15.0, 8.5 Hz, H-23), 3.59 (1H, ddd, *J* = 11.0, 6.5, 4.0 Hz, H-3), 1.02 (3H, d, *J* = 6.5 Hz, H-21), 0.84 (3H, d, *J* = 6.5 Hz, H-27), 0.81 (3H, t, *J* = 5.0 Hz, H-29), 0.80 (3H, d, *J* = 6.0 Hz, H-26), 0.79 (3H, s, H-19), 0.54 (3H, s, H-18); ^13^C NMR (125 MHz, chloroform-*d*) *δ* 139.6 (C-8), 138.3 (C-22), 129.5 (C-23), 117.5 (C-7), 71.1 (C-3), 55.9 (C-17), 55.2 (C-14), 51.3 (C-24), 49.5 (C-9), 43.3 (C-13), 40.9 (C-20), 40.3 (C-5), 39.5 (C-12), 38.0 (C-4), 37.2 (C-1), 34.3 (C-10), 32.0 (C-25), 31.5 (C-2), 29.7 (C-6), 28.6 (C-16), 25.5 (C-28), 23.1 (C-15), 21.6 (C-11), 21.5 (C-21), 21.2 (C-27), 19.1 (C-26), 13.1 (C-19), 12.3 (C-29), 12.1 (C-18); ESI-MS *m*/*z* 413.0 [M+1]^+^.

### 4.4. Cell Culture and Chemicals

The mouse skeletal muscle cell line C2C12 was obtained from the American Type Culture Collection (Manassas, VA, USA) and cultured in Dulbecco’s Modified Eagle Medium (DMEM; Cellgro, Manassas, VA, USA) containing 1% penicillin/streptomycin (P/S) and 10% fetal bovine serum (FBS) at 5% CO_2_ at 37 °C. Rat insulinoma cells, INS-1, were obtained from Biohermes (Shanghai, China) and cultured in Roswell Park Memorial Institute 1640 medium (Cellgro) containing 11 mM D-glucose, 0.05 mM 2-mercaptoethanol, 2 mM L-glutamine, 1% P/S, 1 mM sodium pyruvate, 10 mM HEPES, and 10% FBS at 5% CO_2_ at 37 °C.

### 4.5. Cell Viability Assay

C2C12 cells and INS-1 cells were incubated with treatment samples in 96-well plates overnight. After termination of treatment, cell viability was determined using the Ez-Cytox cell viability detection kit from Daeil Lab Service Co. (Seoul, Korea) according to the manufacturer’s instructions. Ez-Cytox reagent was added to each well and incubated for 2 h at 37 °C. The absorbance of the colored product was measured at 490 nm using a PowerWave XS microplate reader (Bio-Tek Instruments, Winooski, VT, USA).

### 4.6. Glucose Uptake Assay

C2C12 cells were cultured in DMEM, supplemented with 1% P/S and 10% FBS until 95% confluency and transferred to DMEM containing 1% P/S, 2% horse serum, 10% FBS, and 2% bovine serum albumin in the absence or presence of *A. pseudoglehnii*, its fractions, and α-Spinasterol for 16 h. Glucose uptake activity in C2C12 myotubes was examined using a 2-(N-(7-nitrobenz-2-oxa-1,3-diazol-4-yl) amino)-2-deoxyglucose (2-NBDG) uptake assay kit (Sigma-Aldrich, St. Louis, MO, USA) in accordance with the manufacturer’s instructions.

### 4.7. GSIS Assay

GSIS was measured using a rat insulin ELISA kit (Gentaur, Shibayagi Co. Ltd., Gunma, Shibukaw, Japan) in accordance with the manufacturer’s instructions. INS-1 cells were incubated with Krebs–Ringer bicarbonate HEPES buffer (KRBB; 4.8 mM KCl, 129 mM NaCl, 1.2 mM KH_2_PO_4_, 1.2 mM MgSO_4_, 2.5 mM CaCl_2_, 10 mM HEPES, 5 mM NaHCO_3_, and 0.1% BSA, pH 7.4) containing α-Spinasterol, gliclazide (positive control), nifedipine (L-type Ca^2+^ channel blocker), Bay K 8644 (L-type Ca^2+^ channel activator), diazoxide (K^+^ channel activator), or glibenclamide (K^+^ channel blocker) in 12-well plates for 2 h; this was followed by incubation in KRBB containing 2.8 mM and 16.7 mM glucose for 1 h. After termination of treatment, GSIS was assessed using a rat insulin ELISA kit in accordance with the supplier’s instructions.

### 4.8. ADP/ATP Ratio Assay

INS-1 cells were incubated with KRBB containing α-Spinasterol in 12-well plates for 2 h, followed by incubation in KRBB containing 2.8 mM and 16.7 mM glucose for 1 h. After termination of treatment, the ADP/ATP ratio assay kit was used to examine the ADP/ATP ratio in cell lysates (Sigma-Aldrich), according to the supplier’s instructions.

### 4.9. Western Blot Analysis

C2C12 cells and INS-1 cells were incubated with α-Spinasterol in 6-well plates overnight. After termination of treatment, cellular proteins were extracted in RIPA buffer (Cell Signaling, Danvers, MA, USA) on ice for 20 min. Equal amounts of protein were resolved by their molecular size using sodium dodecyl sulfate-polyacrylamide gel electrophoresis and transferred to nitrocellulose membranes [37]. The nitrocellulose membranes were probed with the relevant primary antibodies against P-IRS-1 (# 2385S, 1:1000, Cell Signaling), IRS-1 (# 2382S, 1:1000, Cell Signaling), P-AMPK (# 2531S, 1:1000, Cell Signaling), AMPK (# 5832S, 1:1000, Cell Signaling), GLUT-4 (# 2213S, 1:1000, Cell Signaling), P-IRS-2 (# 07-1517, 1:1000, Sigma-Aldrich), IRS-2 (# 3089S, 1:1000, Cell Signaling), PPARγ (# 2435S, 1:1000, Cell Signaling), PDX-1 (# 5679S, 1:1000, Cell Signaling), followed by horseradish peroxidase-(HRP)-conjugated anti-rabbit secondary antibodies (Cell Signaling) for 1 h on ice, and signals were detected using enhanced chemiluminescence reagent (GE Healthcare UK Limited, Buckinghamshire, UK) for 5 min at room temperature. The results were detected using a chemiluminescence system (FUSION Solo, PEQLAB Biotechnologie GmbH, Erlangen, Germany).

### 4.10. Statistical Analysis

Statistical significance was performed using one-way analysis of variance (ANOVA), with the Bonferroni correction for multiple comparisons. All data represent the mean ± standard error of the mean (S.E.M.). Statistical significance was set at * *p* < 0.01 and *^#^* *p* < 0.05. All analyses were performed using SPSS Statistics, ver. 19.0 (SPSS Inc., Chicago, IL, USA).

## 5. Conclusions

The present study demonstrated that α-Spinasterol isolated from whole plants of *A. pseudoglehnii* improve glucose uptake in C2C12 cells and induce GSIS in INS-1 cells in vitro. In addition, α-Spinasterol-treated C2C12 cells showed increased protein expression of GLUT-4, as well as elevated levels of phosphorylated IRS-1 and AMPK, which were demonstrated to possess important roles in mediating these effects. In addition, α-Spinasterol enhanced GSIS in INS-1 cells by altering the intracellular ATP/ADP ratio and modulating K^+^ and Ca^2+^ channels. α-Spinasterol-treated INS-1 cells demonstrated increased protein expression of PPARγ and PDX-1 and phosphorylation levels of IRS-2, exhibiting important roles in mediating these effects. Further studies, including animal experiments, are necessary to comprehensively elucidate additional mechanisms of action. Accordingly, these results indicate that treatment with α-Spinasterol may be useful in developing novel anti-T2D strategies.

## Supplementary Materials

The following supporting information can be downloaded at: https://www.mdpi.com/article/10.3390/plants11050658/s1, Figure S1: LC-ESI-MS spectrum of α-Spinasterol; Figure S2: ^1^H NMR spectrum (500 MHz, chloroform-*d*) of α-Spinasterol; and Figure S3: ^13^C NMR spectrum (125 MHz, chloroform-*d*) of α-Spinasterol.
