# Supplementary material for: Dual Beneficial Effects of α-Spinasterol Isolated from Aster pseudoglehnii on Glucose Uptake in Skeletal Muscle Cells and Glucose-Stimulated Insulin Secretion in Pancreatic β-Cells
Source: Plants (Basel). 2022 Feb 28;11(5):658. doi: 10.3390/plants11050658 (PMC8912510; doi:10.3390/plants11050658)

Supplementary Data

Dual beneficial Effects of α-Spinasterol isolated from *Aster pseudoglehnii* on Glucose Uptake in Skeletal Muscle Cells and Glucose-Stimulated Insulin Secretion in Pancreatic β-Cells

Dahae Lee [1†], Ji-Young Kim [2†], Hak Cheol Kwon [3], Jaeyoung Kwon [3], Dae Sik Jang [2,*] and Ki Sung Kang [1,*]

Figure S1. LC-ESI-MS spectrum of α-spinasterol.

Figure S2. ^{1}H NMR spectrum (500 MHz, chloroform-*d*) of α-spinasterol.

Figure S3. ^{13}C NMR spectrum (125 MHz, chloroform-*d*) of α-spinasterol.

Figure S1. LC-ESI-MS spectrum of α-spinasterol.

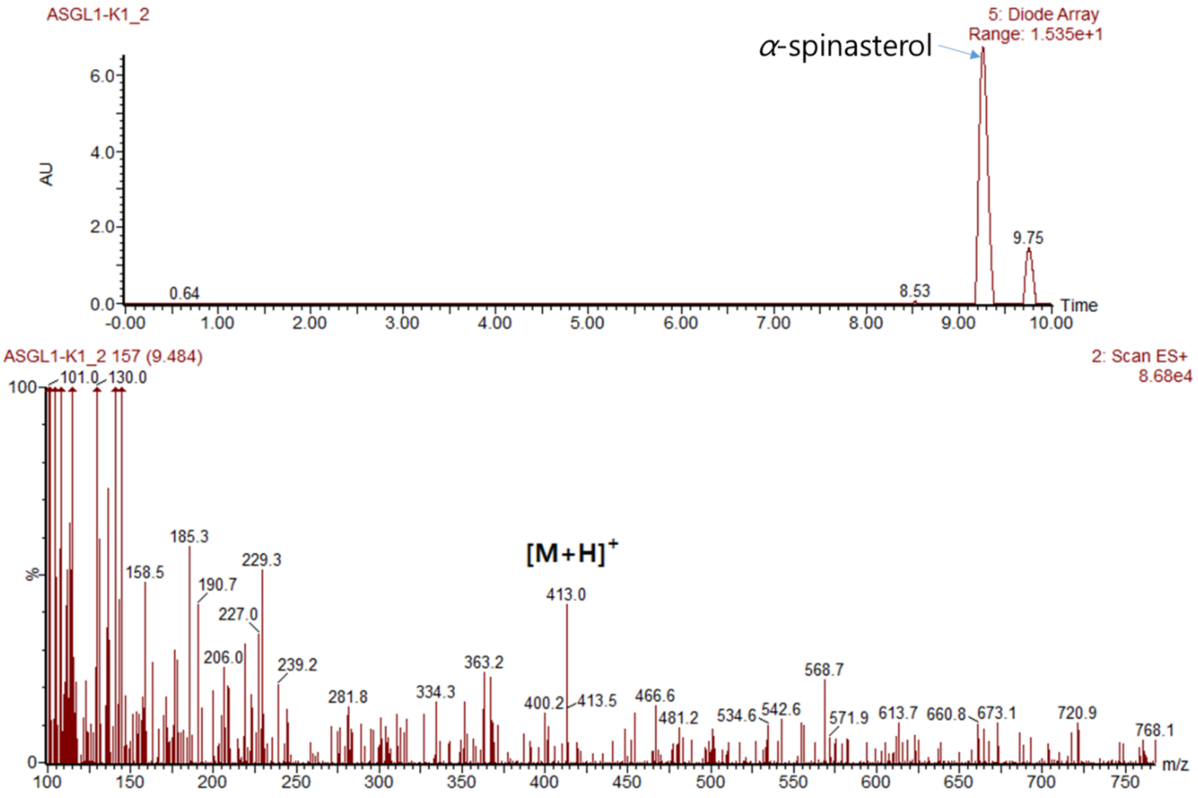

LC-ESI-MS experiment was performed using Waters Acquity UPLC system and Waters Micromass Quattro micro API (Waters, Milford, Massachusetts, USA) with ACQUITY UPLC BEH C18 column (2.1× 50 mm i.d. 1.7 μm, Waters, Milford, Massachusetts, USA).

The mobile phase consisted of water contained 0.1 % formic acid (solvent A) and acetonitrile contained 0.1 % formic acid (solvent B) were carried out using gradient elution at a flow rate 0.2ml/min as follows: 30 % B (0-0.5 min); 30% to 90 % B (0.5-9.5); 100% B (9.5-10 min). The column was equilibrated with initial conditions for one minute before next injections. The injection volume was 3 μl and the column temperature was 25°C.

Figure S2. ^{1}H NMR spectrum (500 MHz, chloroform-*d*) of *α*-spinasterol.

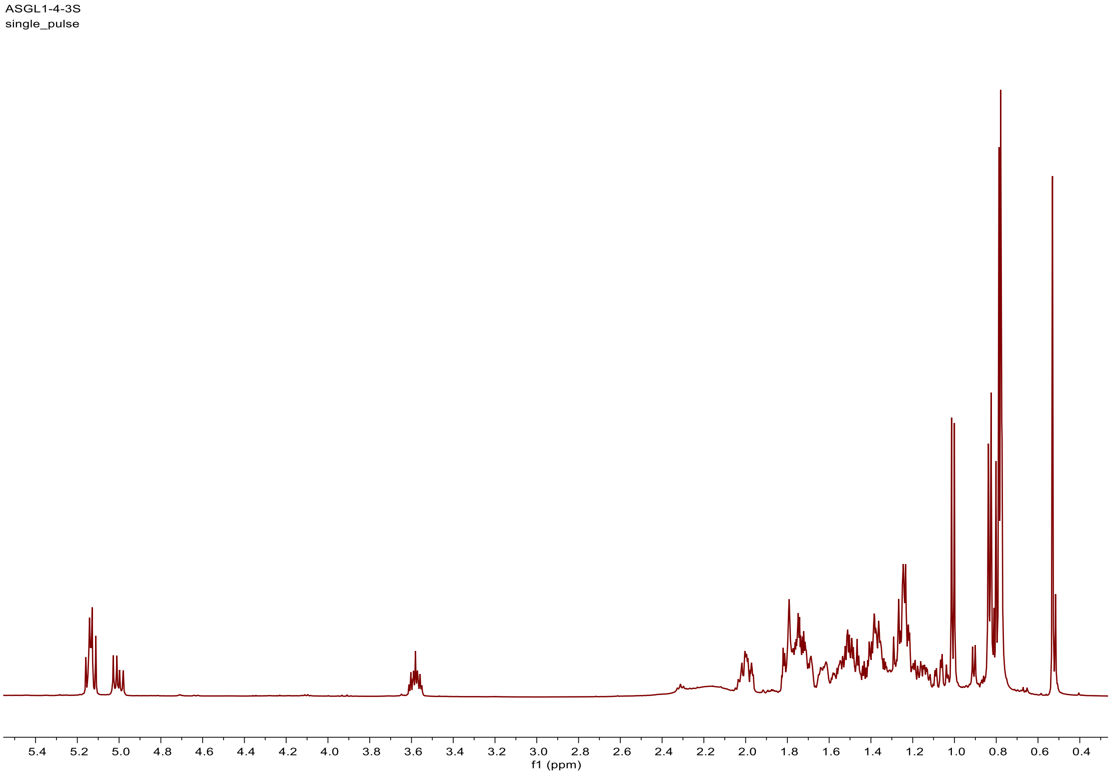

Figure S3. ^{13}C NMR spectrum (125 MHz, chloroform-*d*) of *α*-spinasterol.

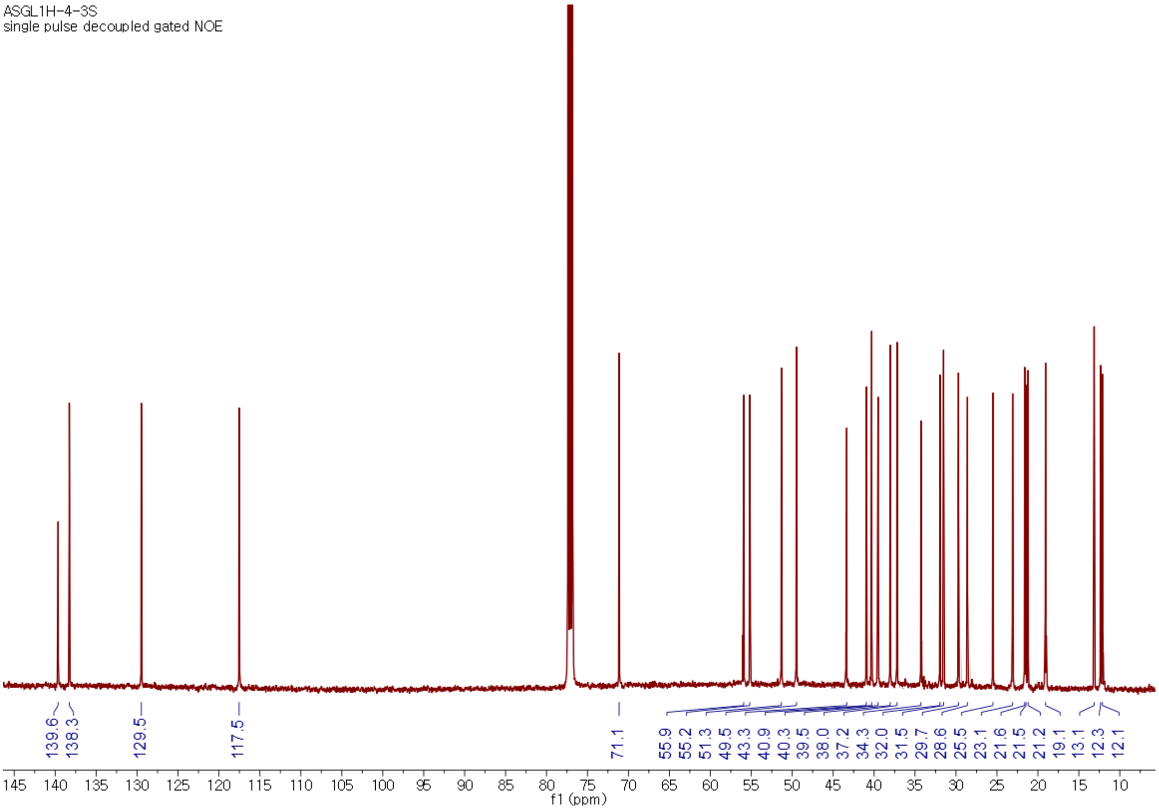

Supplement: Supplementary file 1 [file plants-11-00658-s001.zip › plants-1573873-supplementary.pdf]
